# LPAR5 confers radioresistance to cancer cells associated with EMT activation via the ERK/Snail pathway

**DOI:** 10.1186/s12967-022-03673-4

**Published:** 2022-10-05

**Authors:** Xiao-Ya Sun, Hao-Zheng Li, Da-Fei Xie, Shan-Shan Gao, Xin Huang, Hua Guan, Chen-Jun Bai, Ping-Kun Zhou

**Affiliations:** 1 College of Public Health, Hengyang Medical School, University of South China, Hengyang, 421001 Hunan People’s Republic of China; 2 Department of Radiation Biology, Beijing Key Laboratory for Radiobiology, Beijing Institute of Radiation Medicine, Beijing, 100850 People’s Republic of China

**Keywords:** LPAR5, ERK, Radioresistance, EMT, Radiotherapy

## Abstract

**Background:**

Epithelial-to-mesenchymal transition (EMT) is a critical event contributing to more aggressive phenotypes in cancer cells. EMT is frequently activated in radiation-targeted cells during the course of radiotherapy, which often endows cancers with acquired radioresistance. However, the upstream molecules driving the signaling pathways of radiation-induced EMT have not been fully delineated.

**Methods:**

In this study, RNA-seq-based transcriptome analysis was performed to identify the early responsive genes of HeLa cells to γ-ray irradiation. EMT-associated genes were knocked down by siRNA technology or overexpressed in HeLa cells and A549 cells, and the resulting changes in phenotypes of EMT and radiosensitivity were assessed using qPCR and Western blotting analyses, migration assays, colony-forming ability and apoptosis of flow cytometer assays.

**Results:**

Through RNA-seq-based transcriptome analysis, we found that LPAR5 is downregulated in the early response of HeLa cells to γ-ray irradiation. Radiation-induced alterations in LPAR5 expression were further revealed to be a bidirectional dynamic process in HeLa and A549 cells, i.e., the early downregulating phase at 2 ~ 4 h and the late upregulating phase at 24 h post-irradiation. Overexpression of LPAR5 prompts EMT programing and migration of cancer cells. Moreover, increased expression of LPAR5 is significantly associated with IR-induced EMT and confers radioresistance to cancer cells. Knockdown of LPAR5 suppressed IR-induced EMT by attenuating the activation of ERK signaling and downstream Snail, MMP1, and MMP9 expression.

**Conclusions:**

LPAR5 is an important upstream regulator of IR-induced EMT that modulates the ERK/Snail pathway. This study provides further insights into understanding the mechanism of radiation-induced EMT and identifies promising targets for improving the effectiveness of cancer radiation therapy.

**Supplementary Information:**

The online version contains supplementary material available at 10.1186/s12967-022-03673-4.

## Introduction

Radiotherapy is one of the most commonly used cancer therapy measures of the past century. It is sometimes used alone but is now more frequently applied in combination strategies with other therapeutic methods, including chemotherapy and immunotherapy [1, 2]. Ionizing radiation (IR) kills the cells in a dose-dependent manner through indirect and direct effects, and double-strand breaks (DSBs) are the main molecular events induced by IR to trigger the DNA damage response and cell death signaling [3]. Radioresistance of cancer cells and side effects of normal tissue injuries are two main barriers hindering the clinical application of radiotherapy. A series of reports indicated that epithelial-mesenchymal transition (EMT) is one of the most important endogenous factors promoting the radioresistance of cancer cells and the development of normal tissue fibrosis [4–9].

EMT is a biological process that refers to the transformation of epithelial cells into cells with a mesenchymal phenotype under the control of a set of gene expression products. EMT results in reduced adhesion between cells and other epithelial cells and the molecular characteristics of losing epithelial adhesion markers (E-cadherin) and gaining mesenchymal markers such as N-cadherin, vimentin and fibronectin [10]. During cancer progression, cancer cells originating from epithelial cells can develop both mesenchymal and epithelial characteristics, exhibiting a hybrid E/M phenotype in a process known as partial EMT [11]. This phenotype of partial EMT is thought to confer radioresistance/chemoresistance [12], metastasis [13, 14], and immune escape [15] to tumors and generate circulating tumor cells [16, 17] and cancer stem cells [14]. EMT can endow tumors with radioresistance, and in turn, irradiation may further prompt EMT programming of cancer cells, ultimately increasing radioresistance or metastatic progression.

Accumulating evidence suggests that IR-induced EMT accelerates the malignant progression of tumors in the course of radiotherapy, resulting in increased invasion, migration, radioresistance and chemoresistance in esophageal, cervical, breast, lung and liver cancers, ultimately leading to tumor recurrence and treatment failure [4, 18–22].

EMT programs are regulated by multiple signaling pathways, including those triggered by transforming growth factor-β (TGF-β), epidermal growth factor (EGF), and their associated signaling drivers, such as Notch, Wnt, NF-κB, ERK, Hedgehog, and PI3K/Akt. These signaling molecules can be activated by ionizing radiation and are involved in the IR-induced EMT of cancer cells and normal tissue cells [23–25], *e.g.,* pulmonary epithelial cells [26–28]. Due to the genetic diversity of cancer cells and complexity of radiation-induced cellular responses, the understanding of the mechanism of IR-induced EMT is still limited. Lysophosphatidic acid receptor 5 (LPAR5) is a recently discovered member of the LPAR family. All LPAR members belong to the G protein-coupled receptor (GPCR) family [29]. These 7-transmembrane GPCRs couple to one or more of the four classes of heterotrimeric G-proteins. All LPAR members signal through at least two of the four Gα subunit families (Gα12/13, Gαq/11, Gαi/o and GαS) [30, 31]. Activated G proteins recruit downstream secondary messengers to regulate cell proliferation and cell migration [32]. It has been shown that LPAR plays an important role in the occurrence and development of cancer. It is abnormally expressed in a variety of tumors and mediates tumor growth, invasion and metastasis [33]. LPAR5 is upregulated in papillary thyroid carcinoma (PTC), and downregulation of LPAR5 decreases the proliferation and migration phenotype via the PI3K/Akt pathway [34]. LPAR5 promotes PTC metastasis and tumorigenesis by activating the PI3K/AKT pathway, and LPAR5 regulates the expression of EMT-related proteins, thereby affecting invasion and migration [35]. Knockout of LPAR5 significantly reduces melanoma-derived lung metastases in mice [36]. Upregulation of the LPAR5 gene with an abnormally unmethylated state has been reported to enhance cell proliferation and motility in rat liver-derived hepatoma RH7777 and in lung-derived adenocarcinoma RLCNR cells [37]. However, LPAR5 has also been reported to negatively regulate cell motility and invasive activity in human osteosarcoma cells [38]. Although LPAR5 signaling is clearly associated with cancer initiation, progression and metastasis, whether and how LPAR5 is involved in IR-induced EMT and radiosensitivity of cancer cells remain unclear.

In this study, we constructed the responsive transcriptome of 4 Gy γ-ray irradiated HeLa cells by RNA-seq to identify key molecules involved in early responses to ionizing radiation. LPAR5 was found to be significantly depressed by irradiation. Our results demonstrate that LPAR5 acts as a key regulator of IR-induced EMT in tumor cells through the ERK/Snail pathway.

## Results

### Transcriptome-wide RNA-seq assay to identify early responsive targets determining the sensitivity of cancer cells to radiotherapy

**Fig. 1:**
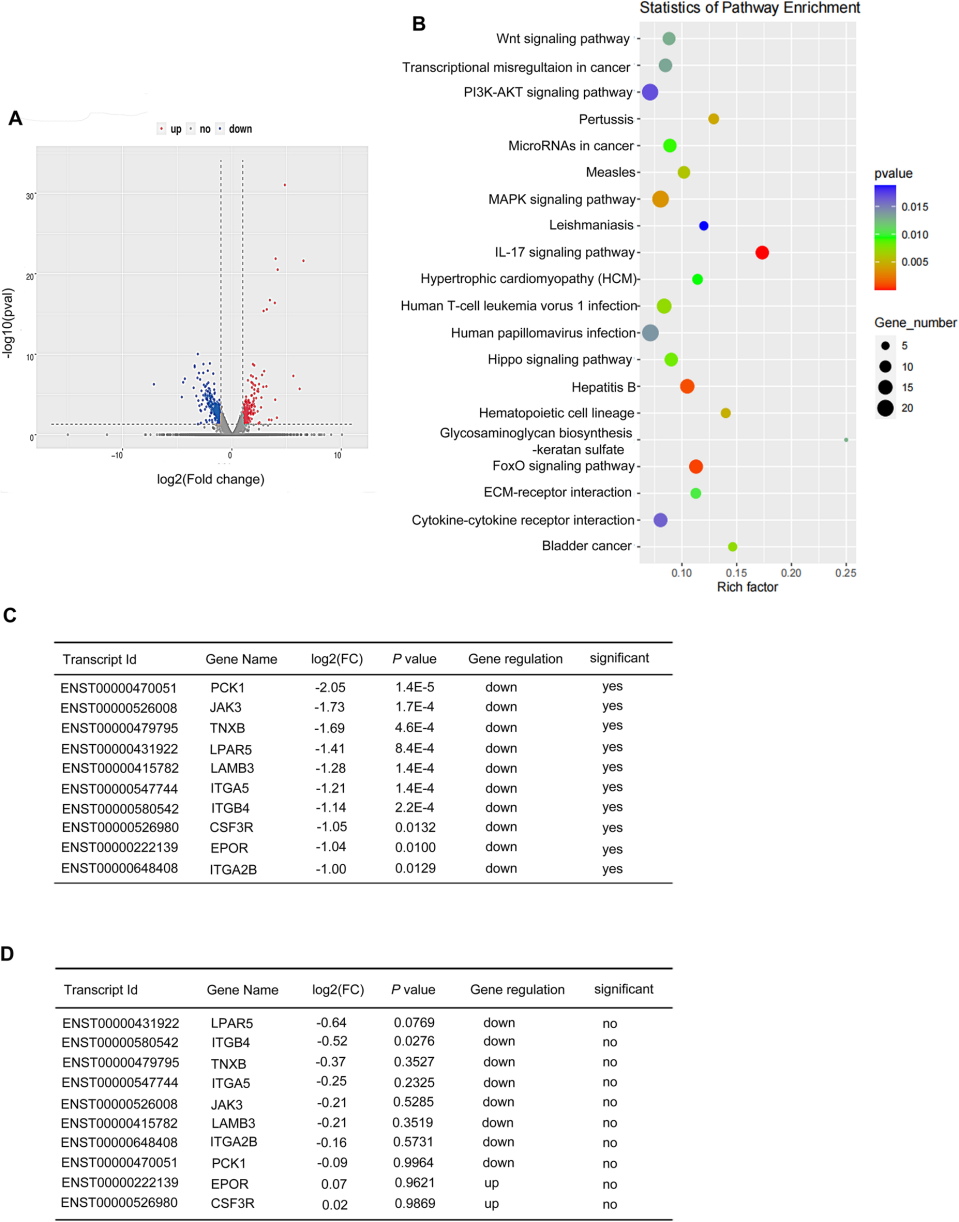

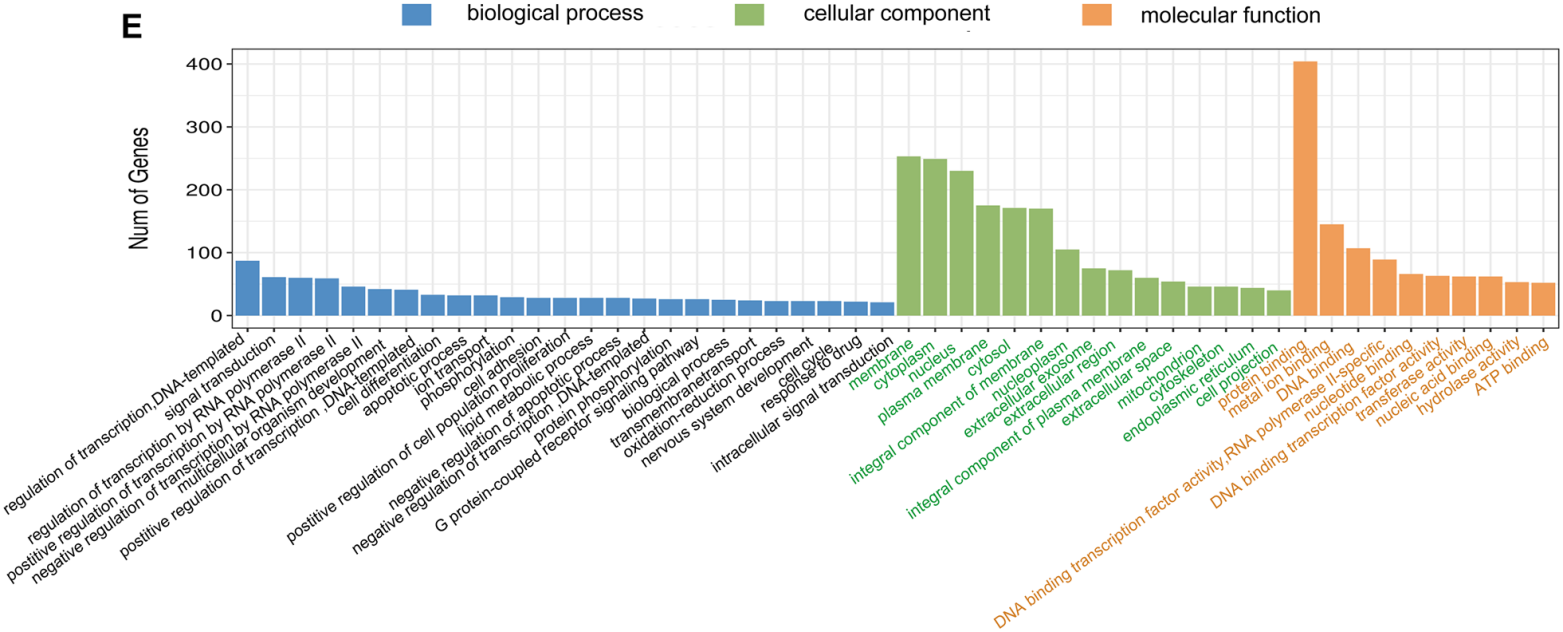
Transcriptome-wide RNA-seq assay to identify early responsive targets for determining the sensitivity of cancer cells to radiotherapy.** A** Scatter plot showing RNA expression changes of genes in HeLa cells 2 h after irradiation compared to unirradiated cells. **B** KEGG analysis of differentially expressed genes in HeLa cells 2 h after irradiation compared to unirradiated cells. **C** Top 10 significantly downregulated PI3K pathway genes in HeLa cells 2 h after 4 Gy irradiation compared to unirradiated cells. **D** Analysis of the 10 genes in panel C in HeLa cells 2 h after 4 Gy irradiation compared to 0.5 h after 4 Gy irradiation. **E** GO analysis of differentially expressed genes in HeLa cells 2 h after irradiation compared to unirradiated cells

To reveal the patterns of radiation affecting messenger RNA profiles for identifying the early responsive targets determining the sensitivity of cancer cells to radiotherapy, we performed transcriptome-wide RNA-sequencing (RNA-seq) assays in HeLa cells, including at 0.5 h and 2 h after 4 Gy γ-ray irradiation. We obtained sequencing information for 29,529 genes in total. In the 0.5 h vs. con group, there were 372 differentially expressed genes with statistical significance compared to the control cells, including 312 downregulated genes (83.8%) and 60 upregulated genes (16.1%) (Additional file 1: Fig. S1A and Additional file 2: Fig. S2A). The same analysis method found that in the 2 h vs. con group, 492 (59%) genes had a downward trend, and 345 (41%) genes had an upward trend (Fig. 1A and Additional file 1: Fig. S1B). Therefore, we chose genes with differentially downregulated expression as the research object. KEGG enrichment analysis showed that in the 0.5 h vs. con group, differentially expressed genes were enriched in tumor development-related pathways, such as the PI3K-AKT signaling pathway, PPAR signaling pathway and NOD-like receptor signaling pathway (Additional file 1: Fig. S2B). In the 2 h vs. con group, KEGG enrichment analysis showed that differentially expressed genes were enriched in the PI3K-AKT signaling pathway, MAPK signaling pathway and Wnt signaling pathway (Fig. 1B). Compared with the KEGG analysis of sequencing at two different time points, we found that the PI3K-Akt signaling pathway was significantly changed, so we chose the genes related to the PI3K-Akt signaling pathway as the selection range. We found the top 10 genes with downregulated differential changes in the 2 h vs. con group and compared the differential changes in these genes in the 2 h vs. 0.5 h group (Fig. 1C, D and Additional file 3: Fig. S3). Among them, only the LPAR5 gene was downregulated most significantly, indicating that it may have a radiation time effect relationship. GO analysis showed that the differentially expressed mRNAs in irradiated HeLa cells are involved in a variety of biological processes, including signal transduction, apoptotic processes, and G protein-coupled receptor signaling pathways, are distributed in various cellular components (membrane, cytoplasm, plasma membrane, nucleus), and play roles in multiple molecular functions (protein binding, metal ion binding, DNA binding) (Fig. 1E and Additional file 2: Fig. S2C). At the same time, we focused on the correlation of the G protein-coupled receptor signaling pathway in the GO analysis results. It has been reported that the LPAR5 protein is also involved in the regulatory mechanism of this pathway. Therefore, we chose LPAR5 as the study object for the next validation step.

### Bidirectional alterations in LPAR5 expression induced by radiation

**Fig. 2:**
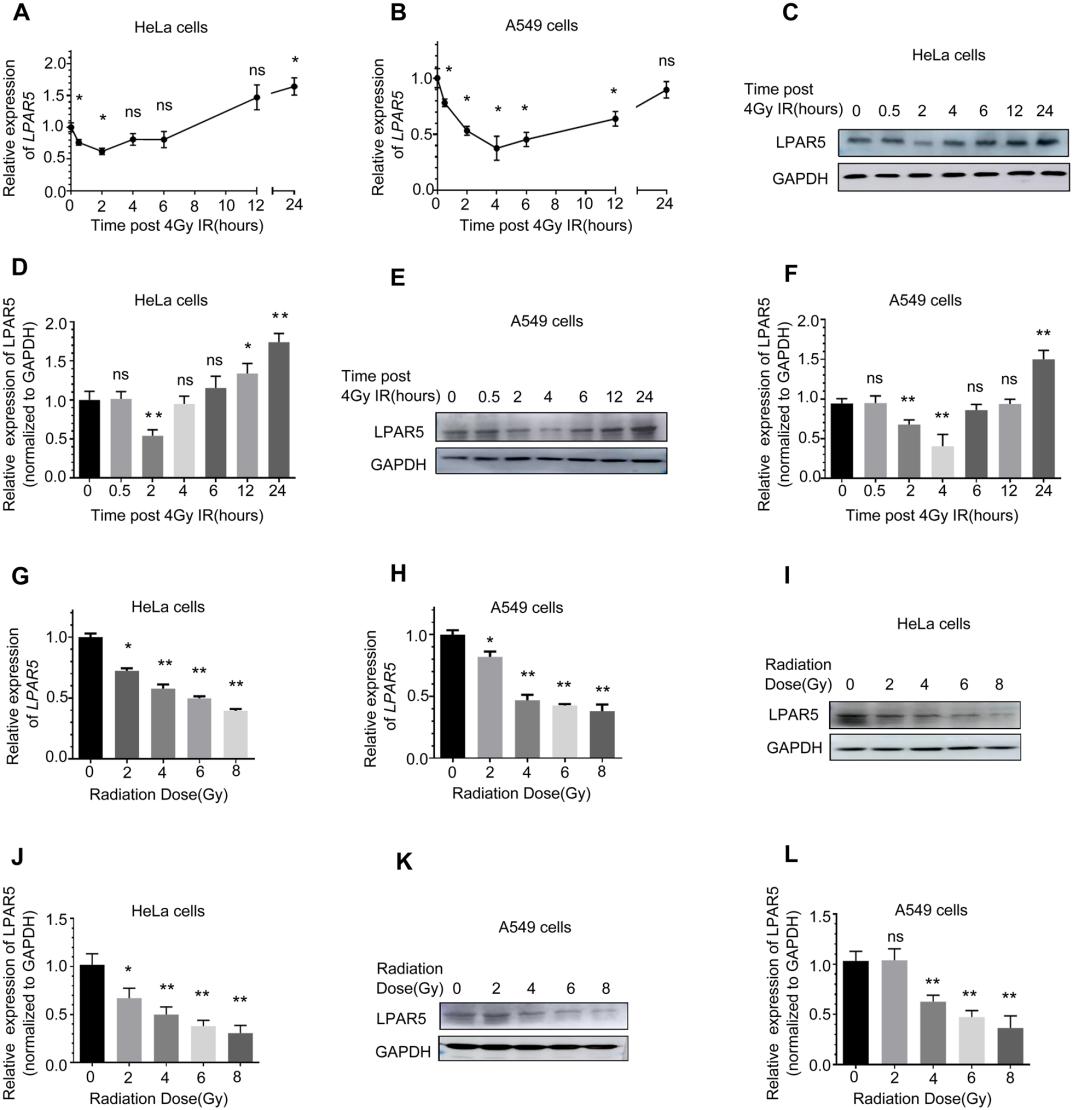
Alterations in LPAR5 expression induced by radiation. **A**, **B** mRNA levels of *LPAR5* in HeLa cells **A** and A549** B** cells at different time points after 4 Gy irradiation, as detected by qPCR. **C**–**F** Protein levels of LPAR5 in HeLa cells **C**, **D** and A549 cells **E**, **F** at different time points after 4 Gy irradiation, as detected by Western blotting and quantified. (G and H) mRNA levels of *LPAR5* in HeLa cells **G** and A549 cells **H** 2 h after irradiation with different doses, as detected by qPCR. **I**–**L** Protein levels of LPAR5 in HeLa cells (I and J) and A549 cells **K**, **L** 2 h after irradiation with different doses, as detected by Western blotting and quantified. Data represent the means ± SDs from three independent experiments. *P < 0.05; **P < 0.01

We performed RT-qPCR analysis and confirmed that the expression of *LPAR5* mRNA was suppressed by 4 Gy γ-ray irradiation in HeLa and A549 cells (Fig. 2). Compared with the unirradiated cells, the RNA expression of *LPAR5* significantly decreased at 2 h and 4 h after 4 Gy irradiation and then gradually recovered (Fig. 2A, B). Western blot analysis also showed that in HeLa cells and A549 cells, the expression of LPAR5 protein decreased within 2–4 h after irradiation and thereafter gradually recovered, even increasing significantly at 24 h after irradiation (Fig. 2C–F). Therefore, ionizing radiation changes the expression of LPAR5 in bidirectional dynamic processes of the early downregulating phase and late upregulating phase. We further confirmed the dose-dependent inhibition of *LPAR5* expression at 2 h after irradiation at the mRNA level detected by RT-qPCR (Fig. 2G , H) and the protein level detected by Western blotting analysis (Fig. 2I–L) in both HeLa (Fig. 2G, I and J) and A549 cells (Fig. 2H, K and L).

### Knockdown of LPAR5 inhibits cancer cell growth and metastasis and promotes apoptosis

**Fig. 3:**
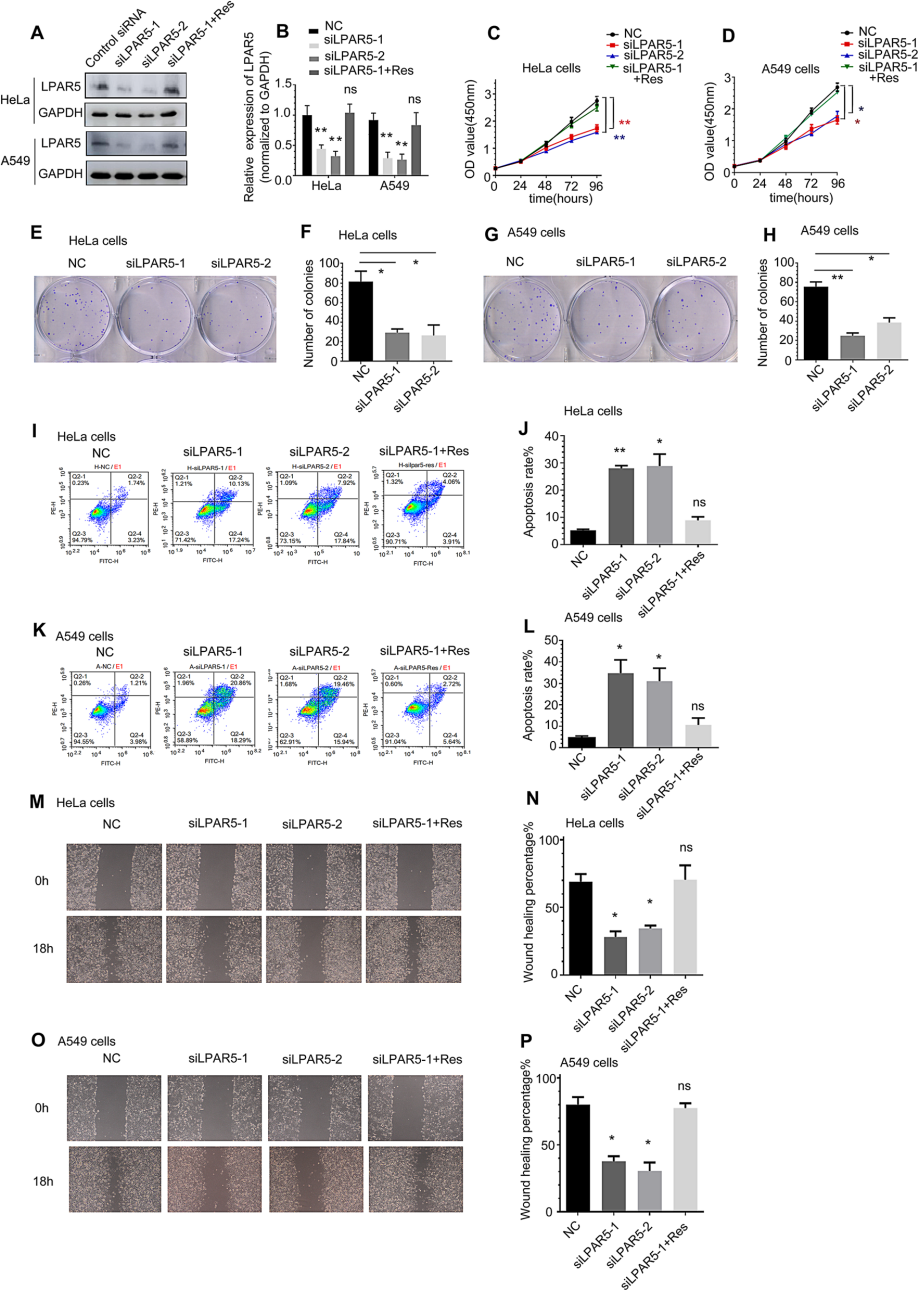
Knockdown of LPAR5 inhibits cancer cell growth and migration and promotes apoptosis. **A**, **B** Knockdown efficiency of LPAR5 mediated by siRNA in HeLa cells and A549 cells, as detected by Western blotting and quantified. **C**, **D** Effects of knocking down LPAR5 on the proliferation of HeLa cells **C** and A549 cells (**D**). **E**–**H** Effects of knocking down LPAR5 on the colony-forming abilities of HeLa cells **E**, **F** and A549 cells (**G**, **H**). **I**–**L** Induction of LPAR5 knockdown on apoptosis in HeLa cells **I**, **J** and A549 cells (**K**, **L**). **M**–**P** Effects of knocking down LPAR5 on the migratory abilities of HeLa cells **M**, **N** and A549 cells (**O**, **P**). Data represent the means ± SDs from three independent experiments. *P < 0.05; **P < 0.01

To explore the effects of LPAR5 on the proliferation phenotype of cancer cells, we knocked down LPAR5 in HeLa cells and A549 cells with specific siRNA molecules (Fig. 3A, B) and found that the proliferation of both cell lines was significantly suppressed (Fig. 3C, D). The suppressed cell growth was rescued by overexpression of exogenous siRNA-resistant LPAR5 vectors. Furthermore, knockdown of LPAR5 significantly suppressed the colony-forming ability of HeLa (Fig. 3E, F) and A549 (Fig. 3G, H) cells. On the other hand, knockdown of LPAR5 promoted the apoptosis of HeLa (Fig. 3I, J) and A549 cells (Fig. 3K, L), which was attenuated by overexpression of exogenous siRNA-resistant LPAR5 vectors. The wound healing assay indicated that knockdown of LPAR5 dramatically inhibited the migratory ability of both HeLa (Fig. 3M, N) and A549 cells (Fig. 3O, P).

### Overexpression of LPAR5 attenuates apoptosis and proliferation inhibition induced by radiation

**Fig. 4:**
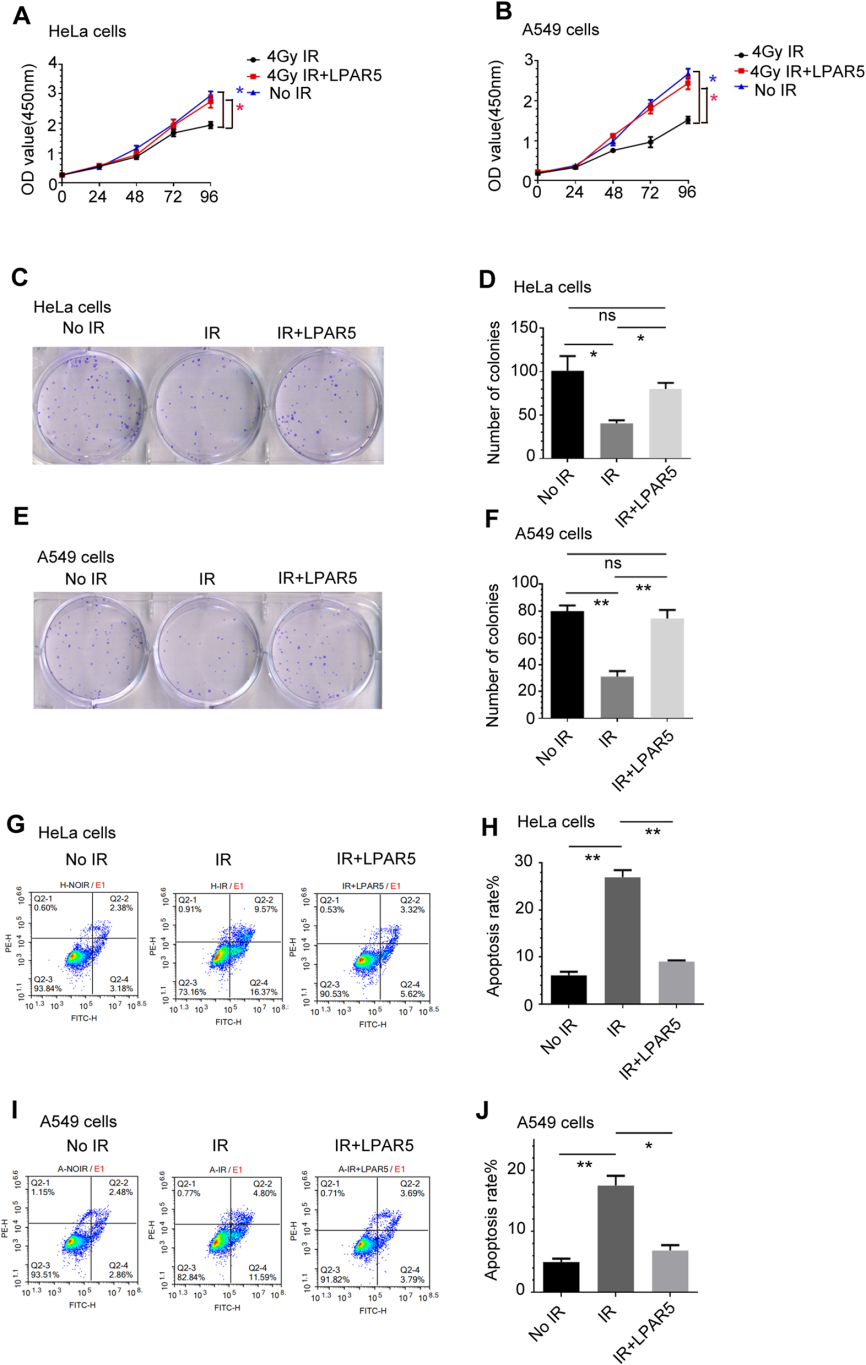
Overexpression of LPAR5 attenuates apoptosis induction and proliferation inhibition by irradiation. **A**, **B** Effects of LPAR5 on proliferation in HeLa **A** and A549 **B** cells upon 4 Gy irradiation. **C**–**F** Effects of LPAR5 on the colony-forming abilities of HeLa (C and D) and A549 (E and F) cells upon 4 Gy irradiation. **G**–**J** Effects of LPAR5 on apoptosis induction in HeLa (G and H) and A549 (I and J) cells upon 4 Gy irradiation. Data represent the means ± SDs from three independent experiments. *P < 0.05; **P < 0.01

To further explore the effects of LPAR5 on IR-induced apoptosis and inhibition of proliferation, we irradiated HeLa and A549 cells with LPAR5. As shown in Fig. 4A, B cell proliferation was suppressed by γ-ray irradiation, which was largely attenuated by overexpressing LPAR5. Similarly, overexpression of LPAR5 significantly attenuated the annihilation of the colony-forming ability of HeLa (Fig. 4C, D) and A549 (Fig. 4E , F) cells after irradiation. We also found that overexpression of LPAR5 significantly reduced the apoptosis induction of HeLa (Fig. 4G, H) and A549 (Fig. 4I, J) cells caused by γ-ray irradiation.

### LPAR5 plays a critical role in radiation-induced EMT

**Fig. 5:**
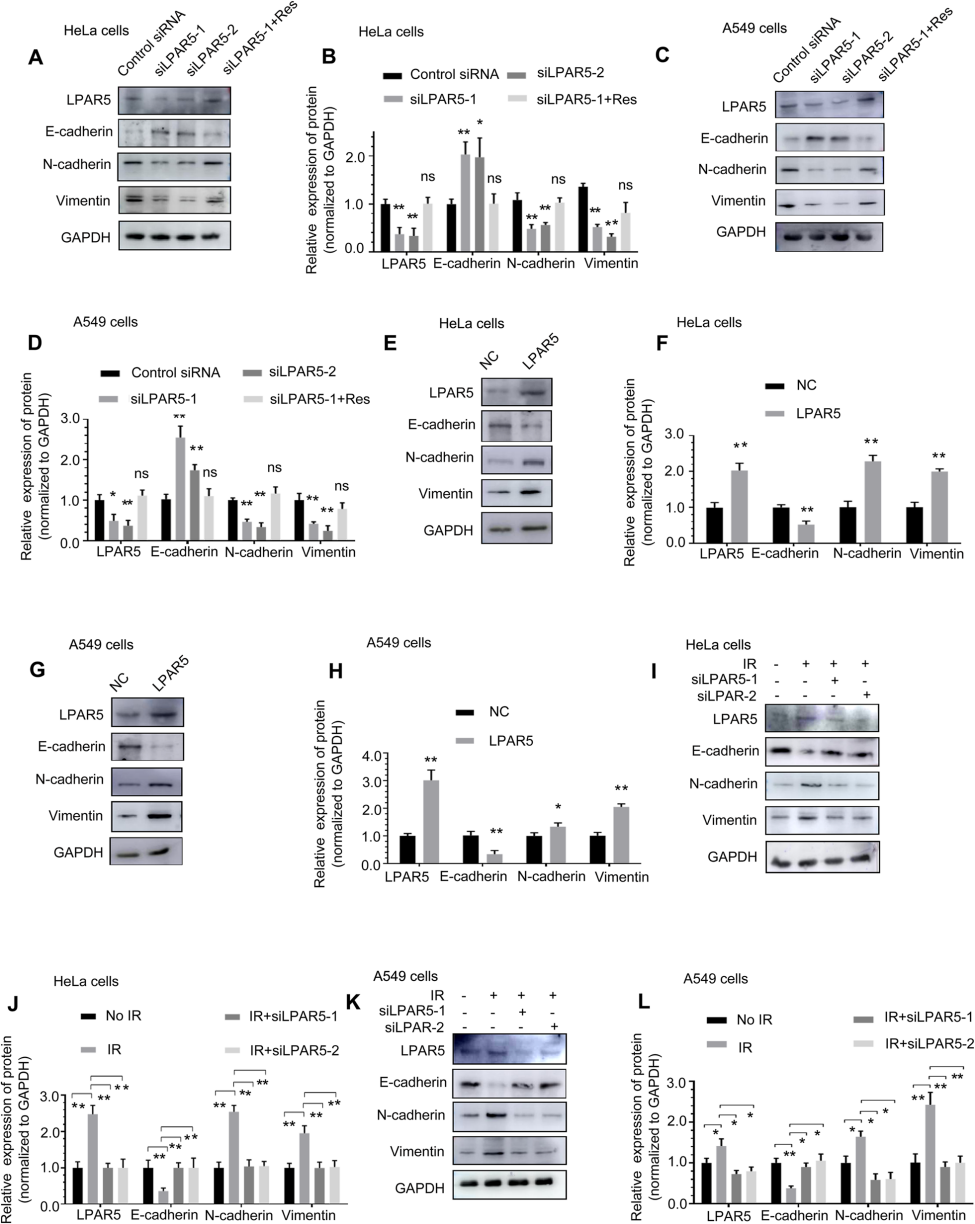
LPAR5 is involved in radiation-induced EMT. **A**–**D** The effect of LPAR5 knockdown on the expression of E-cadherin, N-cadherin and vimentin in HeLa **A**, **B** and A549 **C**, **D** cells, as detected by Western blotting and quantified. **E**–**H** The effect of LPAR5 overexpression on the expression of E-cadherin, N-cadherin and vimentin in HeLa **E**, **F** and A549 **G**, **D** cells, as detected by Western blotting and quantified. **I**–**L** Inhibition of LPAR5 suppression mediated by siRNA altered the expression levels of E-cadherin, N-cadherin and vimentin in HeLa **I**, **J** and A549 **K**, **L** cells induced by 4 Gy irradiation, as detected by Western blotting and quantified. Data represent the means ± SDs from three independent experiments. *P < 0.05; **P < 0.01

According to our data, LPAR5 is an important molecule affecting the migration of HeLa cells and A549 cells. To further clarify the role of LPAR5, we determined its potential action on EMT in cancer cells. Western blot analysis showed that compared with the control group, E-cadherin expression was upregulated in HeLa and A549 cells after knocking down LPAR5, while N-cadherin and vimentin expression was downregulated, which could be rescued by overexpressing siRNA-resistant LPAR5 (Fig. 5A–D). Conversely, overexpressing LPAR5 downregulated E-cadherin expression and upregulated N-cadherin and vimentin expression (Fig. 5E–H). These results indicated that LPAR5 promotes EMT. HeLa and A549 cells underwent EMT at 24 h after 4 Gy irradiation, as demonstrated by decreased E-cadherin expression and increased N-cadherin and vimentin expression (Fig. 5I–L). When LPAR5 was knocked down by specific siRNA in HeLa and A549 cells, IR-induced EMT was inhibited (Fig. 5I–L). The results indicated that LPAR5 plays a positive role in promoting IR-induced EMT.

### LPAR5 promoted radiation-induced EMT through the ERK/Snail axis.

**Fig. 6:**
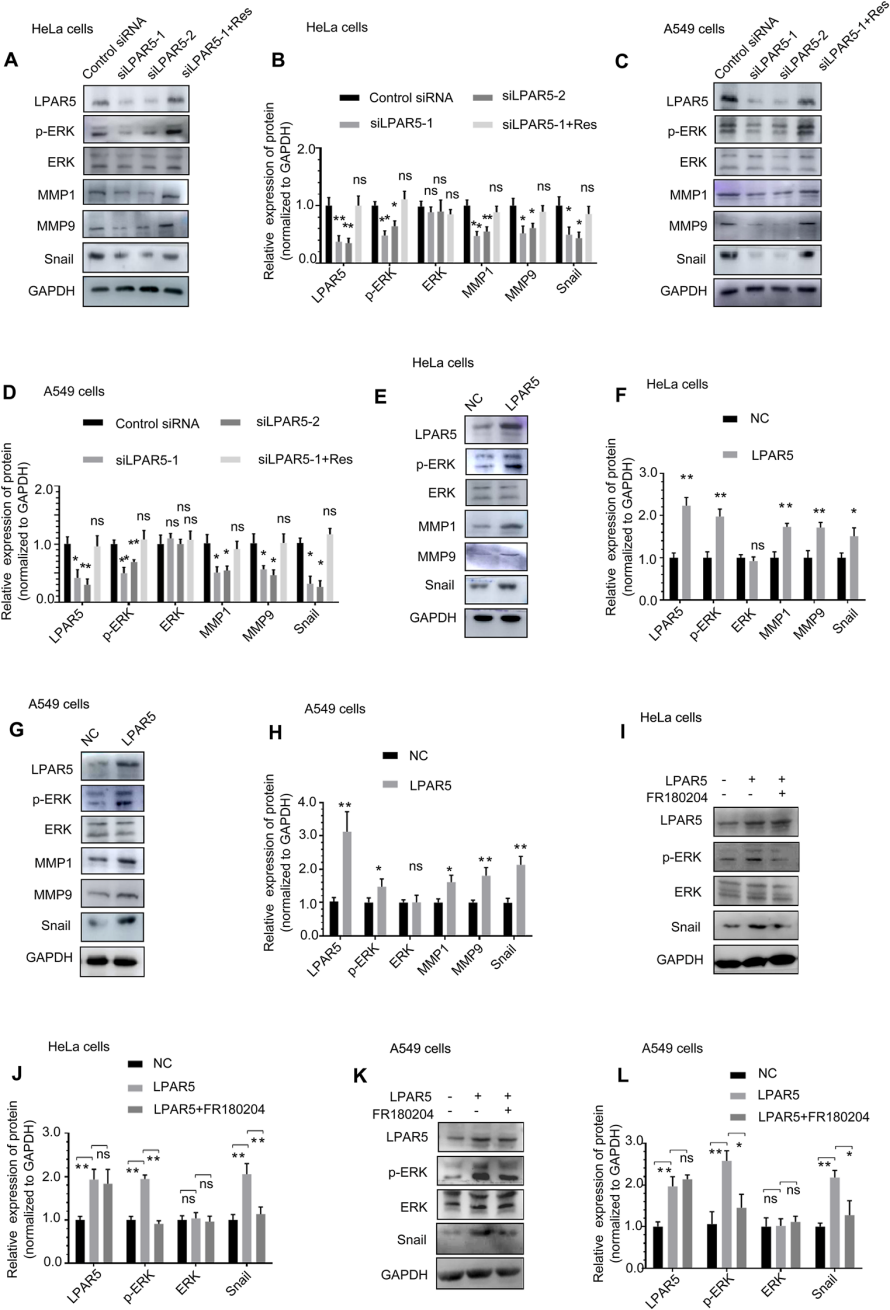

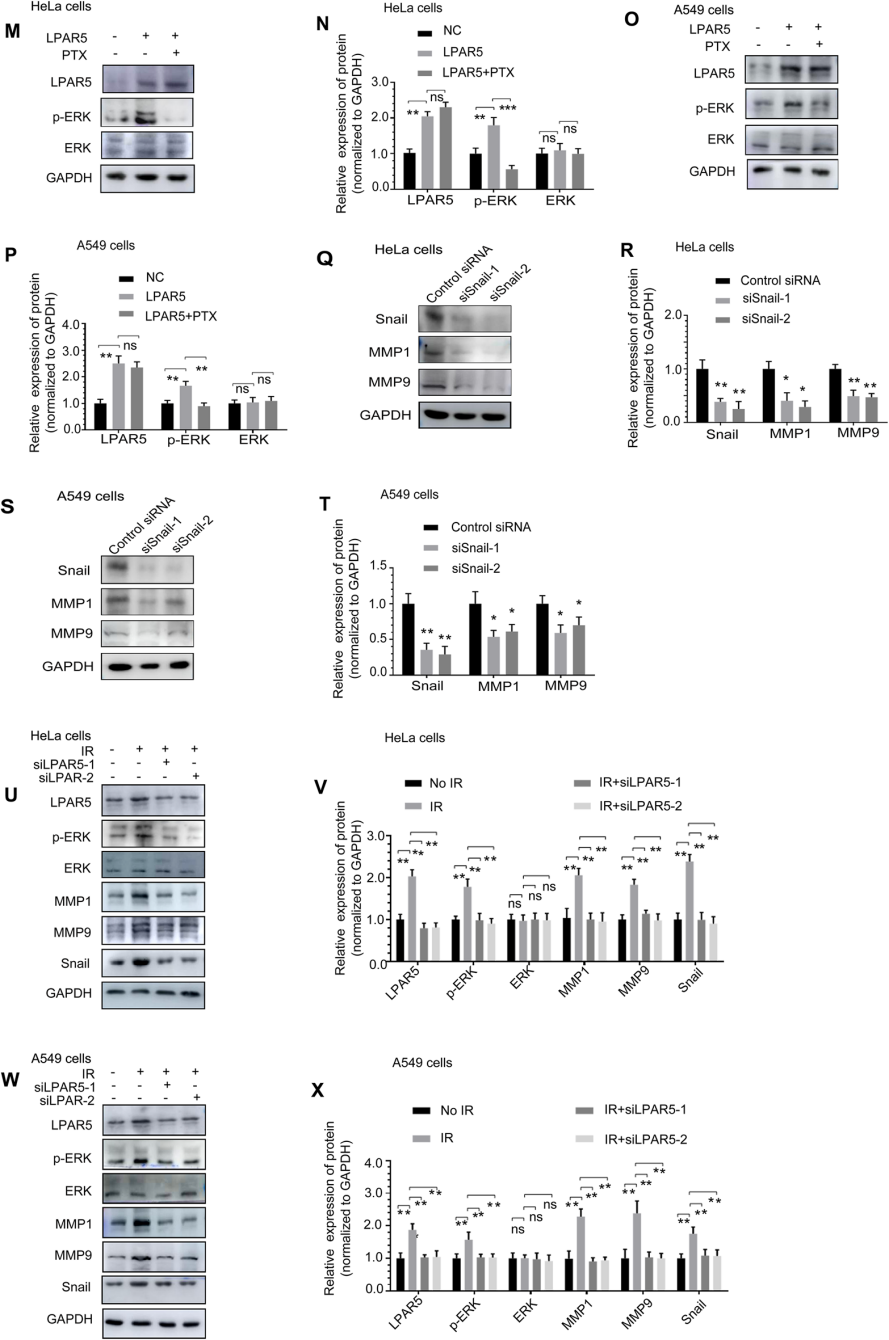
LPAR5 promoted radiation-induced EMT through the ERK/Snail axis. **A**–**D** The effect of LPAR5 knockdown on the expression of p-ERK, ERK, MMP1, MMP9 and Snail in HeLa **A**, **B** cells and A549 **C**, **D** cells, as detected by Western blotting and quantified. **E**–**H** The effect of LPAR5 overexpression on the expression of p-ERK, ERK, MMP1, MMP9 and Snail in HeLa **E**, **F** cells and A549 **G**, **H** cells, as detected by Western blotting and quantified. **I**–**L** Protein expression levels of LPAR5, p-ERK, ERK and Snail in HeLa **I**, **J** cells or A549 **K**, **L** cells overexpressing LPAR5 without or with combined treatment with an ERK inhibitor, as detected by Western blotting and quantified. **M**–**P** Protein expression levels of LPAR5, p-ERK and ERK in HeLa **M**, **N** cells or A549 **O**, **P** cells overexpressing LPAR5 with or without combined PTX treatment, as detected by Western blotting and quantified. **Q**–**T** The effect of Snail knockdown on the expression of MMP1 and MMP9 in HeLa **Q**, **R** cells and A549 **S**, **T** cells, as detected by Western blotting and quantified. **U**–**X** Attenuation of LPAR5 suppression by siRNA on the activation of p-ERK, ERK, MMP1, MMP9 and Snail in HeLa **U**, **V** and A549 **W**, **X** cells induced by 4 Gy irradiation. Data represent the means ± SDs from three independent experiments. *P < 0.05; **P < 0.01

To investigate the molecular pathway by which LPAR5 functions in IR-induced EMT, we tested the effect of LPAR5 on related EMT regulators and transcription factors. Western blot analysis showed that compared with the control group, p-ERK, MMP1, MMP9 and Snail expression was downregulated in HeLa and A549 cells after siRNA-mediated knockdown of LPAR5, while the expression of constitutive ERK was not affected (Fig. 6A–D). These alterations were rescued by overexpression of siRNA-resistant LPAR5. Overexpression of LPAR5 upregulated p-ERK, MMP1, MMP9 and Snail expression but not constitutive ERK expression (Fig. 6E–H). In addition, after pretreatment with the ERK inhibitor FR180204, compared with those in the LPAR5-overexpressing group, the expression levels of p-ERK and Snail were downregulated in HeLa (Fig. 6I, J) and A549 cells (Fig. 6K, L), while the expression of constitutive ERK was not affected. Pertusis toxin (PTX) is known to catalyze the ADP-ribosylation of the Gi subunits of the heterotrimeric G protein, thereby inhibiting the GPCR signaling pathway. After pretreatment with PTX, compared with the LPAR5-overexpressing group, the expression levels of p-ERK were downregulated in HeLa (Fig. 6M, N) and A549 cells (Fig. 6O, P), while the expression of constitutive ERK was not affected. These results suggest that LPAR5 activates the ERK cascade through Gi. Western blot analysis showed that compared with the control group, MMP1 and MMP9 expression was downregulated in HeLa (Fig. 6Q, R) and A549 (Fig. 6S, T) cells after siRNA-mediated knockdown of Snail. These results suggest that LPAR5 can strictly control the expression of Snail, a critical component in EMT programing through the ERK pathway. The expression levels of p-ERK, MMP1, MMP9 and Snail were upregulated in HeLa (Fig. 6U, V) and A549 cells (Fig. 6W, X) 24 h after 4 Gy irradiation and were attenuated by knocking down LPAR5. The expression of LPAR5 protein was significantly increased at 24 h after 4 Gy irradiation, which may contribute to the activation of p-ERK and Snail and consequently promote the EMT program.

## Discussion

Radiotherapy is a common and effective method for the clinical treatment of tumors. However, radioresistance is one of the main challenges for the applications of radiotherapy. At present, a series of studies have pointed out that radioresistance is related to DNA repair capacity, reduced apoptosis, cell cycle status, EMT and other factors, among which EMT is one of the most important factors affecting the sensitivity of cancer cells to ionizing radiation (IR) [39–41]. Studies have shown that IR-induced EMT accelerates the invasion, migration, and radioresistance of various tumor cell lines, including lung, liver, and breast cancer, after radiotherapy. In this study, we irradiated cervical cancer HeLa cells and lung cancer A549 cells and found that 4 Gy ^60^Co γ-irradiation induced EMT, characterized by decreased expression of the epithelial marker E-cadherin and enhanced expression of the mesenchymal markers N-cadherin and vimentin. To our surprise, the expression of LPAR5 decreased in a dose-dependent manner for a short time at 2 ~ 4 h after irradiation and then recovered, even increasing significantly 24 h after irradiation. We observed that knockdown of LPAR5 can significantly inhibit the proliferation and migration of tumor cells, while high expression of LPAR5 can increase the proliferation and inhibit apoptosis of tumor cells after irradiation. Similar to this study, researchers have demonstrated that LPAR5 plays an important role in melanoma invasion and metastasis [42]. Knockdown of LPAR5 promotes thyroid cancer (PTC) cell apoptosis and reduces proliferation through the PI3K-Akt pathway [34].

Through this study, we found that LPAR5 is an important molecule affecting the migration of cervical cancer cells and lung cancer cells and is significantly associated with IR-induced EMT. Many studies have pointed out that LPA and its receptors play an important role in the process of tumor cell migration. The LPAR family triggers EMT and tumor progression through crosstalk with downstream signaling pathways, such as the NF-κB, MAPK/ERK, PI3K/AKT, TGF-β and Wnt/β-catenin pathways, and other oncogenic signaling pathways [43–45]. However, there are few studies on the role of LPAR5 in tumor progression. We found that knockdown of LPAR5 significantly inhibited the IR-induced decrease in epithelial markers and elevation of mesenchymal markers, and overexpression of LPAR5 in HeLa cells and A549 cells significantly promoted EMT. LPAR5 had a significant effect on the EMT phenotype, knockdown of LPAR5 significantly inhibited HeLa cell and A549 cell migration, and the above results suggest that LPAR5 is required for IR-induced EMT. Therefore, our data suggest that LPAR5 regulates IR-induced EMT.

Multiple studies have reported that GPCRs activate the ERK cascade through G protein α subunit (including Gs, Gi, and Gq) and G protein βγ subunit signaling [46–48]. We demonstrate that treatment with PTX, a Gi inhibitor, significantly suppressed the expression of p-ERK induced by high levels of LPAR5. This result suggests that Gi plays a critical role in promoting ERK activation mediated by LPAR5. We show for the first time that LPAR5 can regulate Snail expression through the ERK pathway and mediate IR-induced EMT. Highly expressed LPAR5 upregulates the expression of Snail through the ERK pathway, thereby affecting the expression of MMP1 and MMP9.

The ERK pathway is one of the major oncogenic signals in human cancers because its activation leads to an increase in proliferation, invasion, and metastasis [49–51]. The ERK pathway is often hyperactivated in tumors [52] and acts as an inducer of cell migration and invasion through a Snail-mediated mechanism [53–56]. MMPs can degrade various protein components in the extracellular matrix. At the same time, they can also disrupt the histological barrier of tumor cells and affect tumor migration, invasion, metastasis and angiogenesis [57–60], and MMPs are considered the main proteolytic enzymes in this process. We found that knockdown of LPAR5 significantly inhibited Snail, MMP1, and MMP9 elevation after IR treatment, thereby blocking IR-induced EMT. Based on our results and combined with previous reports and results, we believe that the LPAR5/ERK signaling pathway mediates IR-induced EMT through upregulation of Snail. These findings provide strong evidence for the critical upstream role of LPAR5 in regulating the molecular network associated with IR-induced EMT, which contributes to the increased resistance of cancer cells to radiotherapy.

## Materials and methods

### Cell culture

The cell lines used in this study (human alveolar type II epithelial carcinoma cell line A549 and human cervical cancer cell line HeLa) were maintained in our laboratory. HeLa cells and A549 cells were grown in DMEM (HyClone) containing 10% FBS (HyClone and PAN) and 1% penicillin‒streptomycin. All cells were cultured at 37 °C under 5% CO_2_ in a humidified incubator. Cells were subjected to ^60^Co γ-ray irradiation at a dose rate of 85.69 cGy min^–1^ at room temperature.

### Plasmid, siRNA

The full sequence of LPAR5 was cloned into pcDNA3.1 to generate an overexpression plasmid.

For LPAR5 knockdown, two synthesized duplex RNAi oligos targeting human mRNA sequences from Sigma were used (si-LPAR5-1: 5′- GCAGCUGCAUCUUCCUGAUGCUCAU-3′, si-LPAR5-2: 5′-CGCCUGCACUUGGUGGUCUACAGCU-3′). For Snail knockdown, two synthesized duplex RNAi oligos targeting human mRNA sequences from Sigma were used (si-Snail-1: 5′-CACUCAGAUGUCAAGAAGUTT-3′, si-Snail-2: 5′-CAGAUGUCAAGAAGUACCATT-3′). A scrambled duplex RNA oligo (5′-UUCUCCGAACGUGUCACGU-3′) was used as an RNA control. The cells were transfected using Lipofectamine 2000 reagent (Invitrogen) with vector control, plasmid construct, siRNA negative control (siNC), or siRNAs according to the manufacturer’s instructions.

### RNA extraction and qRT-PCR analysis

Total RNA was isolated using TRIzol. One microgram of RNA was reverse-transcribed into cDNA with ReverTra Ace qPCR RT Master Mix with gDNA Remover (TOYOBO, Code No. FSQ-301) according to the manufacturer’s instructions. Quantitative real-time PCR analysis was performed with 1 μL of cDNA using HUNDERBIRD SYBR qPCR Mix (TOYOBO, Code No.QPS-201). β-actin was used as an endogenous control. β-actin primers used for qRT‒PCR, F: 5′-TTGCTGACAGGATGCAGAAG-3, R: 5′-ACTCCTGCTTGCTGATCCACAT-3′. LPAR5 primers used for qRT‒PCR, F: 5′-GAGGTCTCTGCTGCTGAT-3′; R: 5′-AGAACTGTTGGTTGAGGAG-3′.

### Western blot analysis and antibodies

Cells were ruptured with RIPA buffer containing 5 mM EDTA, PMSF, and phosphatase inhibitor cocktail. Cell extracts were centrifuged for 15 min at 12,000 × *g*, and the supernatants were then collected. Approximately 40 μg of total protein was resolved by SDS–polyacrylamide gel electrophoresis, transferred onto nitrocellulose (NC) membranes, blocked with 5% nonfat milk at room temperature for 2 h, and incubated with primary antibodies. After washing three times, the membrane was incubated with horseradish peroxidase (HRP)-conjugated secondary antibodies (1:4000 dilution) for 1 h at room temperature, and the blot was visualized by using SuperSignal™ West Pico Plus Chemiluminescent Substrate (ThermoFisher Scientific, TL275133). The primary detection antibodies were as follows: anti-E-cadherin (Proteintech, 20,874–1-AP, 1:1000), anti-N-cadherin (CST, D4R1H, 1:1000), anti-vimentin (Abcam, ab8978, 1:1000), anti-GAPDH (Santa Cruz, USA, sc-25778, 1:1000), anti-ERK (Abcam, ab17942, 1:1000), anti-Snail (CST, L70G2, 1:1000), anti-MMP1 (NeoMarbers, 1536P8C7C, 1:1000), and anti-MMP9 (Proteintech, 10375-2-AP, 1:1000). Protein expression was detected with an enhanced chemiluminescent reagent (Thermo, MA, USA).

### Proliferation assay

Cells (1.5 × 10^3^) were seeded into 96-well plates and incubated at 37 °C in a humidified 5% CO_2_ atmosphere. Cellular proliferation was measured with a Cell Counting Kit-8 (DOJINDO, SJ608). Briefly, 10 μL/well CCK8 solution was added at the indicated times, and then, the cells were incubated at 37 °C for 3 h. The absorbance at 450 nm was recorded by a microplate reader.

### Cell Migration Assays

Cell migration was examined by wound-healing assays. After treatment of the cells, scratches were made using sterile 10-μL pipette tips, and bright-field microphotographs were taken at different times. The percentages of cell migration were quantitated by ImageJ software by measuring the width of the cell-free zone immediately after scratching.

### Cell apoptosis analysis

To assess cell apoptosis, after treatment with trypsin, the cells were collected and washed twice with PBS. Then, 4 μL of PI (propidium iodide) and 4 μL of FITC (fluorescein isothiocyanate) (Dojindo, AD10) were added after the cells were pelleted and resuspended in 400 μL of 1 × binding buffer. Apoptosis was detected by flow cytometry after 15 min of incubation in the dark at room temperature.

### RNA sequencing and analysis

Total RNA was extracted using TRIzol reagent (Invitrogen, CA, USA) following the manufacturer's procedure. The total RNA quality and quantity were analyzed using a Bioanalyzer 2100 and RNA 6000 Nano LabChip Kit (Agilent, CA, USA) with RIN > 7.0. Approximately more than 200 µg of total RNA was subjected to isolation of poly(A) mRNA with poly-T oligo attached magnetic beads (Invitrogen). Following purification, the poly(A) mRNA fractions were fragmented into 100-nt oligonucleotides using divalent cations under elevated temperature. Fragments were converted to a final cDNA library in accordance with strand-specific library preparation by the dUTP method. The average insert size for the paired-end libraries was 100 ± 50 bp. Then, we performed paired-end 2 × 150 bp sequencing on an Illumina NovaSeq 6000 platform at LC-BIO Biotech, Ltd. (Hangzhou, China).

Cutadapt and perl scripts were used to remove the reads that contained adaptor contamination, low quality bases and undetermined bases to obtain CleanData. Then sequence quality was verified using fastp. Used the default parameters of HISAT2 to map reads to the genome of *homo sapiens* (Version: v96). We used StringTie to quantify gene expression and performed normalization with FPKM method. The gene difference was analyzed with R package edgeR. Used R package exomePeak performed peak scanning in the whole gene range to obtained information such as the position and length of peak on the genome. These peak were annotated by intersection with gene architecture using ChIPseeker and enriched with GO and KEGG.

### Quantification and statistical analysis

Data are presented as the mean ± SEMs or SDs. Statistical analyses were performed in GraphPad Prism 6 (GraphPad Software, Inc.) The unpaired two-tailed Student’s t test was used to compare differences between two groups with a significance of *P* < 0.05. One-way analysis of variance with multiple comparisons tests was used to compare three or more groups with a significance of *P* < 0.05.

## Supplementary Information

**Additional file 1: Figure S1.** Heatmap plot showing RNA expression changes of genes in HeLa cells 0.5 h **A** and 2 h **B** after irradiation compared to unirradiated cells

**Additional file 2: Figure S2. A** Scatter plot showing RNA expression changes of genes in HeLa cells 0.5 h after irradiation compared to unirradiated cells. KEGG **B** and GO **C** analyses of differentially expressed genes in HeLa cells 0.5 h after irradiation compared to unirradiated cells

**Additional file 3: Figure S3.** Significantly regulated PI3K pathway genes in HeLa cells 2 h after 4 Gy irradiation compared to unirradiated cells

## Data Availability

All data needed to evaluate the conclusions in the paper are presented in the paper and/or in the Supplementary Materials. Additional data related to this paper may be requested from the authors.
