# Supplementary figures and images for: LPAR5 confers radioresistance to cancer cells associated with EMT activation via the ERK/Snail pathway
Source: J Transl Med. 2022 Oct 5;20:456. doi: 10.1186/s12967-022-03673-4 (PMC9533496; doi:10.1186/s12967-022-03673-4)

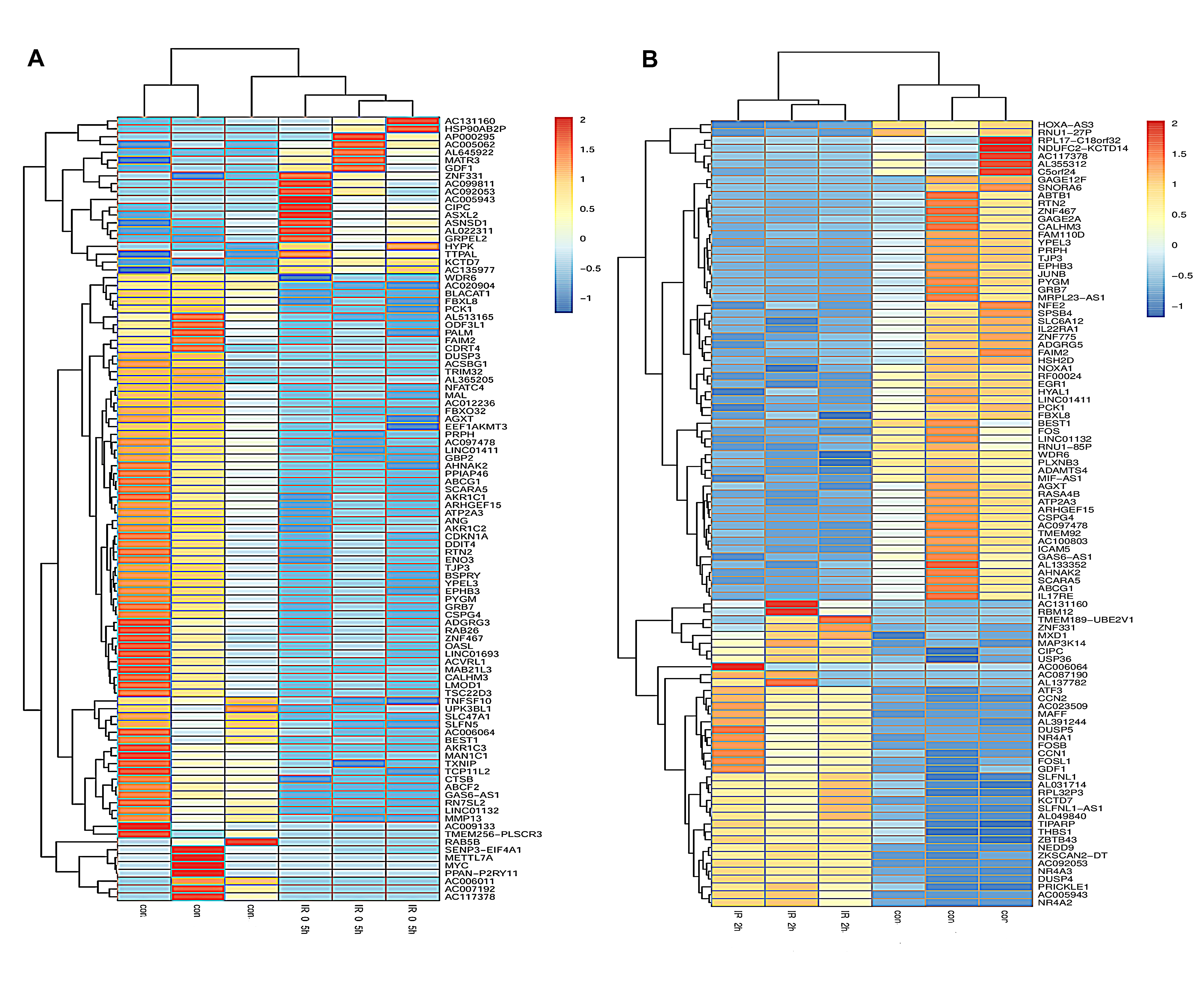

Supplement: Supplementary file 1 — Additional file 1: Figure S1. Heatmap plot showing RNA expression changes of genes in HeLa cells 0.5 h A and 2 h B after irradiation compared to unirradiated cells [file 12967_2022_3673_MOESM1_ESM.tif]

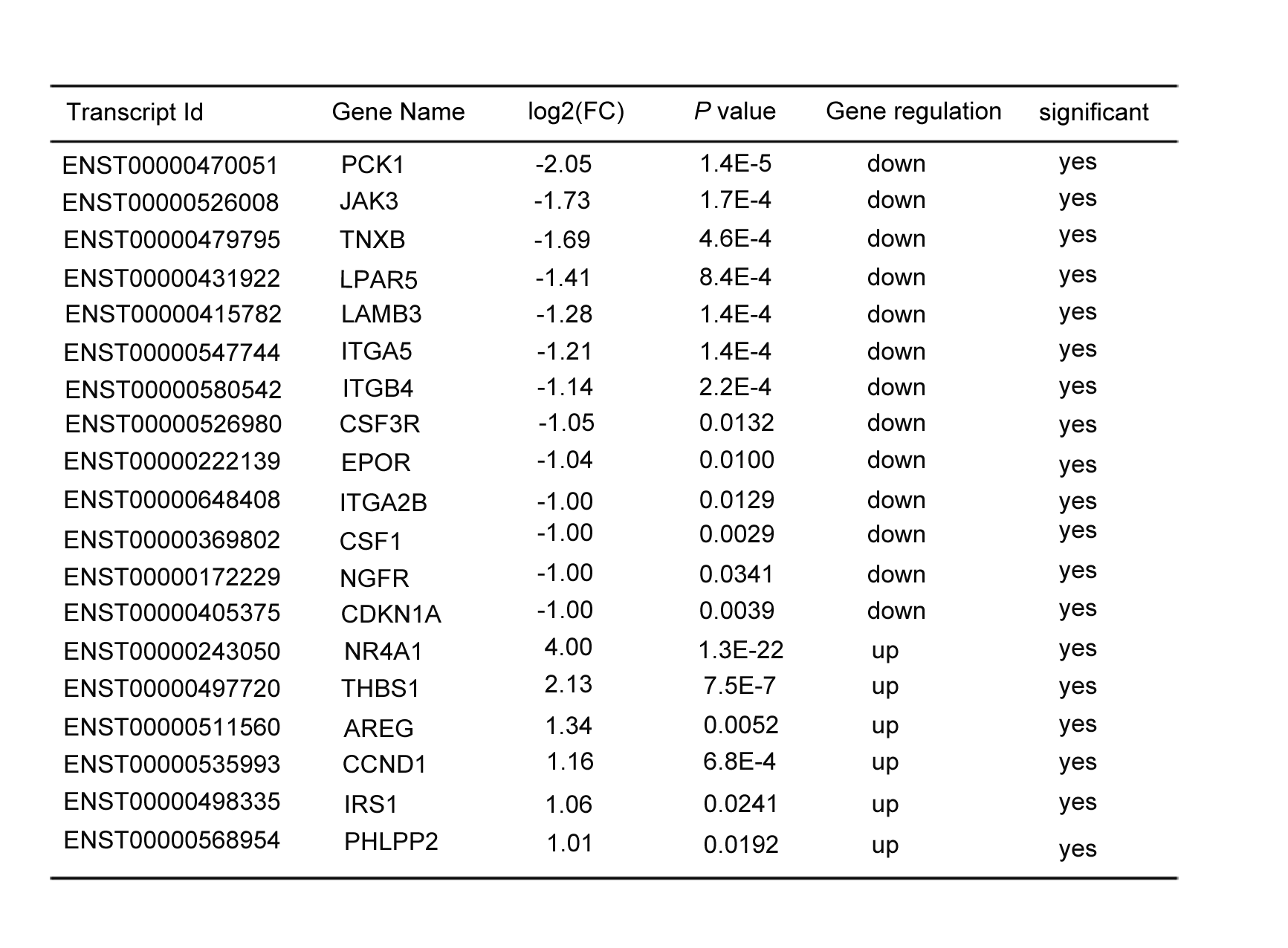

Supplement: Supplementary file 3 — Additional file 3: Figure S3. Significantly regulated PI3K pathway genes in HeLa cells 2 h after 4 Gy irradiation compared to unirradiated cells [file 12967_2022_3673_MOESM3_ESM.tif]
